# Glycemic Response of a Carbohydrate-Protein Bar with Ewe-Goat Whey

**DOI:** 10.3390/nu6062240

**Published:** 2014-06-12

**Authors:** Eirini Manthou, Maria Kanaki, Kalliopi Georgakouli, Chariklia K. Deli, Dimitrios Kouretas, Yiannis Koutedakis, Athanasios Z. Jamurtas

**Affiliations:** 1 Department of Nutrition and Dietetics, Technological Educational Institute of Thessaly, Karditsa 43100, Greece; E-Mails: eirinimanthou@yahoo.gr (E.M.); mrkanaki@gmail.com (M.K.); 2 Department of Physical Education and Sport Science, University of Thessaly, Trikala 42100, Greece; E-Mails: kgeorgakouli@pe.uth.gr (K.G.); delixar@pe.uth.gr (C.K.D.); y.koutedakis@pe.uth.gr (Y.K.); 3 Institute for Research and Technology Thessaly (I.RE.TE.TH), Trikala 42100, Greece; 4 Department of Biochemistry-Biotechnology, University of Thessaly, Larissa 41221, Greece; E-Mail: dkouret@uth.gr; 5 School of Sport, Performing Arts and Leisure, Wolverhampton University, Walshall WS1 3BD, UK

**Keywords:** glycaemic index, glycaemic load, functional food

## Abstract

In this study we examined the glycaemic index (GI) and glycaemic load (GL) of a functional food product, which contains ewe-goat whey protein and carbohydrates in a 1:1 ratio. Nine healthy volunteers, (age, 23.3 ± 3.9 years; body mass index, 24.2 ± 4.1 kg·m^2^; body fat %, 18.6 ± 10.0) randomly consumed either a reference food or amount of the test food both with equal carbohydrate content in two visits. In each visit, seven blood samples were collected; the first sample after an overnight fast and the remaining six at 15, 30, 45, 60, 90 and 120 min after the beginning of food consumption. Plasma glucose concentration was measured and the GI was determined by calculation of the incremental area under the curve. The GL was calculated using the equation: test food GI/100 g available carbohydrates per test food serving. The GI of the test food was found to be 5.18 ± 3.27, while the GL of one test food serving was 1.09 ± 0.68. These results indicate that the tested product can be classified as a low GI (<55) and low GL (<10) food. Given the health benefits of low glycaemic response foods and whey protein consumption, the tested food could potentially promote health beyond basic nutrition.

## 1. Introduction

Functional foods are defined as “foods that, by virtue of the presence of physiologically-active components, provide a health benefit beyond basic nutrition” [1]. As consumer interest in functional dietary items is increasing, there is a need to evaluate all physiologically active components and their role in health promotion [2,3]. Furthermore, their ability to impact metabolic parameters and chronic disease, such as diabetes, cardiovascular disease and cancer is of great interest to health-policy makers and the food industry.

One of the food properties that may enhance health is its glycaemic response, which relates to its glycemic index (GI) and glycaemic load (GL) [4,5]. GI is an estimate of the glycaemic effect of available carbohydrate in a food on blood glucose levels compared to the effect of an equal amount of glucose [6]. GL relates the GI to a specific food serving. Implementation of low glycaemic index (LGI) and/or low glycaemic load (LGL) diets benefit metabolic profiles [4,5,7,8,9] and leads to prevention or slower progression of several types of cancer [10]. Additionally, consuming meals that result in a high glycaemic response may lead to transformation of metabolic pathways, increased appetite, overeating, and increased body mass and body fat [11]. In contrast, both short-term and long-term studies demonstrate that the consumption of LGI or LGL foods, in conjunction with reduced energy intake, assists weight and fat mass reduction [7,8,12,13]. Therefore, foods giving a low glycaemic response may have a profound effect in morbid conditions related to obesity.

In this study we tested a commercially available bar made with organic ingredients. It has a carbohydrate:protein ratio of 1:1 and is considered as a functional product due to its ewe-goat whey content, which has health-enhancing properties [14,15,16]. Given the rapid rise in metabolic disease and obesity in both adults and children [17], there is a great need to better communicate the GI/GL profile of foods to consumers. Therefore, the objective of this study was to determine the GI and GL of a special carbohydrate-protein bar in a healthy population. Results may give further support to the health enhancing properties of this and similar products and enrich the database of GI-labeled and marketed functional food products.

## 2. Experimental Section

### 2.1. Participants

Eleven healthy recreationally active men (*n* = 5) and six women (*n* = 6) were initially recruited. Two women failed to complete the experimental process due to personal reasons. Inclusion criteria included the absence of any clinical symptoms or chronic diseases as determined by a health questionnaire. Exclusion criteria included the presence of gastrointestinal disease or metabolic disease, medication treatment (including nutritional supplements), current pregnancy, lactation or following a specific diet regime. A written informed consent was provided by all participants after they had been informed of all risks, discomforts and benefits involved in the study. The procedures were in accordance to the 1975 Helsinki declaration, and approved by the Institutional Review Board of the University of Thessaly, Greece.

### 2.2. Anthropometric and Physiological Measurement

Participants reported to the laboratory twice. During their first visit, following an overnight fast, they had their body mass, body fat and hydration level assessed using leg-to-leg bioelectrical impedance scales (Tanita BF 522 W, TANITA Corp., Illinois, IL, USA) while lightly dressed and barefooted. Standing height was measured to the nearest 0.5 cm (Stadiometer 208, Seca, Birmingham, UK). Body mass index was calculated using the equation weight (kg)/[height (m)]^2^. Waist and hip circumferences were obtained with measuring tape. Blood pressure was acquired via a manual sphygmomanometer; the lowest of three readings was recorded. Resting heart rate was recorded by short-range telemetry (Polar RS100, Polar Electro Oy, Kempele, Finland). Anthropometric characteristics of all participants appear in Table 1.

**Table 1:** Baseline anthropometric and physiological characteristics of participants (Mean ± SD).

Characteristics	*n* = 9
Age (years)	23.3 ± 3.9
Height (cm)	176.4 ± 13.0
Weight (kg)	75.8 ± 17.7
Body Mass Index (kg/m^2^)	24.2 ± 4.1
Body Fat (%)	18.6 ± 10.0
Hydration (%)	56.3 ± 6.7
Waist/Hip ratio	0.8 ± 0.0
Systolic Blood Pressure (mmHg)	113.9 ± 13.4
Diastolic Blood Pressure (mmHg)	74.2 ± 7.1
Resting Heart Rate (beats·min^−1^)	71.2 ± 4.8

### 2.3. Study Design

According to recommendations by FAO/WHO [18] for the measurement of GI, participants underwent two different trials after overnight fasting separated by at least seven days. In the trials, they consumed in random order either the reference food (250 mL aqueous solution containing 50 g of glucose), or test food of equal carbohydrate content and 250 mL of water. At each visit, blood samples were taken after 12 h fasting at baseline (0 min) and at times 15, 30, 45, 60, 90 and 120 min after the consumption of food. Τhe consumption of the reference or test food was completed within 15 min. No other food or liquid intake was allowed for the next 120 min. Participants were asked to refrain from alcohol, caffeine, and exercise for the two days prior to each trial.

### 2.4. Standard and Test Food

The test food was an organic chocolate- orange flavored bar, which has a carbohydrate:protein:lipid content ratio of 2:2:1. Protein extracts used to prepare the bar derived from ewe-goat milk whey protein. The bar is produced in Greece and is sold under the commercial name “50/50 Feta Bar” by Eatwalk Hellas. Nutritional analysis of the test food is shown in Table 2. The serving size is considered 70 g and all participants consumed an amount of the test food (167 g) that corresponded to 50 g available carbohydrate content. The exact amount of the test food was determined using an analytical balance (Kern ALS 120-4, Balingen, Germany). An aqueous glucose solution containing 50 g of glucose was used as the standard food (Top Star 50, Toplabs, Portugal).

**Table 2:** Nutritional analysis of the test food and portion percentage of the average daily dietary values.

Nutritional Analysis Variables	Per 100 g	Per Portion 70 g	Daily Dietary Values % (70 g)
Energy (kcal)	409.1	286.3	14.3
Protein (g)	30.8	21.6	43.2%
Whey protein (g)	14.3	10.01	43.2%
Total Carbohydrate (g)	30.2	21.1	7.7%
Sugar (g)	19.7	13.8	15.5%
Fiber (g)	5.2	3.6	13.1%
Fat (g)	15.6	10.9	15.9%
Saturated Fat (g)	7.0	4.9	21.2%
Unsaturated Fat (g)	8.6	6.0	-
Polyols (g)	6.0	4.2	-
Sodium × 2.5 (mg)	52	36.4	3.9%
Potassium (mg)	27.5	19.2	-

### 2.5. Blood Collection and Analyses

A flexible catheter was inserted into an antecubital vein and blood was drawn at 0, 15, 30, 45, 60, 90 and 120 min. Blood (7 mL) was drawn at each time point into ethylenediamine tetra-acetic acid (EDTA) tubes which were immediately placed on ice. Following each blood sampling, the catheter was flushed with 2–3 mL saline to prevent blood clotting. A portion (1–2 mL) of the blood collected was used to determine the parameters of the complete blood count measured by an automatic biochemical analyzer (Mythic 18 Orphée, Orphée Medical, Geneva, Switzerland). The remaining blood was centrifuged at 4 °C, 1370 g for 10 min for separation of plasma in a refrigerated centrifuge (Heraeus Biogigure Primo, Thermo Scientific, Waltham, MA, US). Then the collected supernatant was transferred to Eppendorf tubes (Eppendorf TM, Sarstedt AG & Co., Nümbrecht, Germany). The samples were stored at −80 °C and thawed only once before analysis. Plasma glucose concentration was determined using a biochemical analyzer (Clinical Chemistry Analyzer Z 1145-Zafiropoulos Diagnostica, Koropi, Greece).

### 2.6. Dietary Analyses

In order to ensure that the last meal preceding the trials did not affect glycaemic response results, participants were instructed to record their evening meal prior to their first trial; this food intake was replicated the evening before the second trial [19]. Each participant was provided with a written set of guidelines for monitoring dietary consumption and a record sheet for recording food intake. Diet records were subsequently analyzed using the computerized nutritional analysis system DietSpeak^©^ (DietSpeak Hellas, Athens, Greece).

### 2.7. Calculation of GI-Statistical Analyses

GI was calculated with the method of incremental area under the curve (iAUC) ignoring the area under baseline. Specifically, F:R were calculated, where F is the mean iAUC for a group of participants after consuming the test food and R is the mean iAUC for the same group of participants after consuming the reference food [18]. Then the following equation was used to determine the GI of the product [6,18,19]:

GI = iAUC (50 g available carbohydrates of the test food)/iAUC (50 g carbohydrates of the reference food) × 100 (1)

GL of one serving was calculated as follows:

GL = [GI × available carbohydrate in one serving (g)]/100 (2)

**Figure 1:**
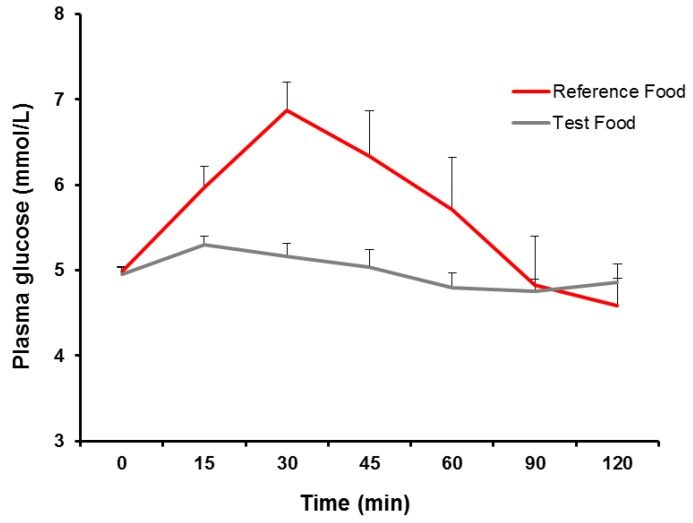
Plasma concentration of glucose during postprandial periods after the reference and test food ingestion (Mean ± SEM).

Baseline values in the two trials were compared by paired *t*-test. *p*-Values were considered significant at *p* < 0.05. Results are presented as (Mean ± SD) apart from GI, GL values and Figure 1 (Mean ± SEM).

## 3. Results

Complete blood count test values obtained during the two trials are presented in Table 3. All values were within the normal range, indicating that the present volunteers were healthy. Fasting glucose values were 4.95 ± 0.26 mmol/L and 4.98 ± 0.14 mmol/L (*p* > 0.05) for the test and reference food trial, respectively. These values were within the normal range (<5.5 mmol/L blood plasma).

**Table 3:** Baseline complete blood count values for individuals participating in the reference and test food trial (Mean ± SD).

Complete Blood Count Variables	Standard Food	Test Food	Normal Range
White Blood Cells (10^3^/μL)	6.4 ± 1.4	6.2 ± 1.0	4.0–12.0
Lymphocytes (10^3^/μL)	2.4 ± 0.7	2.4 ± 0.2	1.0–5.0
Mononuclear Cells (10^3^/μL)	0.6 ± 0.2	0.6 ± 0.1	0.1–1.0
Granulocytes (10^3^/μL)	3.4 ± 0.9	3.2 ± 0.9	2.0–8.0
Lymphocytes (%)	36.7 ± 6.5	39.4 ± 6.6	25.0–50.0
Monocytes (%)	10.3 ± 2.7	9.5 ± 1.8	2.0–10.0
Granulocytes (%)	53.0 ± 5.8	51.1 ± 7.3	50.0–80.0
Red Blood Cells (10^6^/μL)	4.7 ± 0.7	4.7 ± 0.5	4.0–6.2
Hemoglobin (g/dL)	12.9 ± 1.4	13.5 ± 1.5	11.0–18.0
Hematocrit (%)	38.7 ± 3.3	40.1 ± 3.0	35.0–55.0
Mean Corpuscular Volume (μm^3^)	84.2 ± 12.6	86.8 ± 11.0	80.0–100.0
Average Amount of Hemoglobin in Red (Pg)	28.1 ± 4.5	29.3 ± 4.3	26.0–34.0
Mean Corpuscular Hemoglobin Concentration (g/dL)	33.3 ± 0.9	33.7 ± 1.4	31.0–3.5
Erythrocyte Distribution Width (%)	12.4 ± 1.4	11.9 ± 1.3	10.0–16.0
Platelets (10^3^/μL)	243.1 ± 34.2	232.4 ± 47.5	150–400
Mean Platelet Volume (μm^3^)	8.8 ± 0.3	8.8 ± 0.3	7.0–11.0
Platelet Count (%)	0.2 ± 0.0	0.2 ± 0.0	0.2–0.5
Platelet Distribution Width (%)	16.1 ± 4.6	16.4 ± 4.7	10.0–18.0

Mean total energy intake of the evening meal preceding each trial for the nine participants was 628 ± 393.7 kcal. Mean total protein, fat and carbohydrate content of the meal were 24.8 ± 17 g, 29.6 ± 24.8 g and 61.4 ± 38.4 g, respectively. Mean fiber content of the meal was 6.1 ± 5.4 g.

The GI value of the test food resulting from the analyses was 5.18 ± 3.27 and the GL value was 1.09 ± 0.68 g per 70 g serving. The glucose curves after reference and test food ingestion are shown in Figure 1.

## 4. Discussion

The purpose of this study was to determine the glycaemic response of a special carbohydrate-protein bar in a healthy population. The calculated GI and GL of the test food (5.18 ± 3.27 and and 1.09 ± 0.68, respectively) classify it in the group of LGI/LGL food products [12,19]. This could be partly attributed to its functional ingredients as whey protein and fiber. Whey protein has been shown to decrease postprandial glycaemic response in a dose dependent manner through glucose and non-glucose dependent pathways [20,21]. Whey ingestion can exert insulinogenic effects postprandially, while co-ingestion with carbohydrates has a synergistic effect on insulin response [22]. In a study, addition of whey to a common meal (mashed potatoes and meatballs) reduced postprandial blood glucose excursion by 21% [22]. The glycaemic response of the tested food therefore could have been influenced by its high protein content, which was 43% of the average daily dietary value per portion. In addition the equally high proportion of carbohydrates in that bar may have reinforced the insulinotropic effects of whey.

Carbohydrates that resist digestion such as fiber may also substantially lower postprandial glycaemia and has been shown to reduce GI of a product or meal [5]. The presence of fiber in a food may induce prolonged digestion and increased colonic fermentation, which result to moderation of glycaemic responses postprandially [23]. High fiber content of the tested food (5.2 g, approximately 13% of the average daily dietary value per portion), which is equal to a medium sized fruit with skin, may also have contributed to its low GI. Although high fiber content reduces palatability of a product, it may add to the metabolic merits of a LGI/LGL diet. According to its properties the tested bar could be used as part of an LGI/LGL diet. Low glycaemic response diets are proposed as a means to favorably influence physiologic parameters implicated in conditions such as obesity, diabetes mellitus and risk of coronary heart disease [4,5,7,8,9]. A metanalysis by Livesey and colleagues [5] has shown evidence that fasting blood glucose is reduced following the consumption of LGI/LGL foods, especially in individuals with fasting blood glucose concentrations in excess of 5 mmol/L. There is increasing evidence to show that LGI/LGL diets have the potential to protect against diabetes type II and improve glycaemic profiles by reducing insulin secretion and protein glycosylation [9,24]. In contrast, consumption of HGI foods results in postprandial hyperglycaemia and hyperinsulinemia [11,12]. Therefore, a functional food product, as the one we tested, characterized by sweet taste but low glycaemic response could be ideally used against sweet food cravings of type I and II diabetics [25]. It is believed that metabolic syndrome sufferers may also benefit by consumption of LGI/LGL foods as they can induce favorable changes in low density lipoprotein and blood triglycerides, and maintain or slightly improve high density lipoprotein levels [5,9].

Diets that produce a high glycaemic response are blamed for hyperinsulinemia, insulin resistance or reduced glucose tolerance, which seem to be positively correlated with increased risk of several types of cancer [10] and systemic inflammation [26]. In contrast, LGI/LGL food products are considered of superior biological and health value [5]. As obesity has become a major consideration, LGI/LGL diets have been suggested to promote higher fat oxidation and prolonged satiety, which may be conducive to body fat and body mass reduction [13,14]. Also, there is scientific evidence to support the notion that whey protein beneficially affects satiation and satiety [27]; therefore it may help reduce obesity and its co-morbidities. A single serving (70 g) of the tested bar provides 286 kilocalories (kcal), thus, it could be part of a weight loss plan without the side effects of other sweetened food products.

In relation to exercise nutrition, data concerning the GI of food consumed before, after and during exercise are inconclusive. Most studies show that consuming LGI carbohydrate before exercise results in a favourable metabolic profile during exercise, but with limited positive effect on actual performance [28]. No research has indicated that consumption of HGI foods prior to exercise negatively affects exercise performance [28,29]. In a study, consumption of LGI *vs.* HGI carbohydrate during 24 h of recovery after endurance exercise resulted in 7 out of 8 participants running longer in the LGI trial when subsequent exercise was performed in the fasted state [30]. Authors attributed their findings to the higher rate of fat oxidation and a higher concentration of plasma free fatty acids observed during subsequent exercise. Inconclusive findings overall suggest that individuality may be more significant concerning glycaemic response and performance; therefore more properly designed studies should be conducted.

The tested bar may present additional health benefits due to its whey protein content. This protein provides protection and/or improvement in chronic disease such as cardiovascular diseases [14], bacterial infections [31], cancer [32] and osteoporosis [33]. Whey protein is also rich in sulfur-containing cysteine and methionine, which contribute to the enhancement of antioxidant defense through intracellular conversion to glutathione [14]. Its high concentration in branched-chain amino acids plays a key role in protein metabolism which contributes to muscle hypertrophy [34], while it may enhance exercise performance by reducing muscle damage [35] and incidence of overtraining [36]. A similar food product but with lower protein content has been previously investigated in relation to its anti-inflammatory [37] and antioxidant response [38] to exercise. Participants either consumed four servings of the test food or an isocaloric placebo before they undertook 2 h moderate intensity cycling exercise followed by a very intense exercise session leading to exhaustion. While performance was not affected, results indicated that markers of lipid peroxidation (thiobarbituric acid reactive substances) as well as pro-inflammatory markers (interleukin-6 and C-reactive protein) were significantly reduced in the test food trial [37,38].

This study, although carefully conducted is not without limitations. The number of participants may be characterized as limited although very close to the sample number suggested by current literature in order to provide useful results [19]. However, two to three times more subjects would greatly improve precision. Moreover, the time given to subjects to consume the bar was fifteen minutes given its reduced chewability compared to the glucose drink. Therefore, it is highly likely that the prolonged time of ingestion flattened glucose curves in both reference and test food trials [19]. Finally, a catheter was inserted and venous blood was taken throughout the study to avoid frequent punctures of the subject. Even though literature indicates [19] that glucose values might differ when venous blood is used as compared to capillary; we assume that blood sampling did not affect our results since the measurement of GI in the test and standard foods was performed utilizing venous blood.

## 5. Conclusions

The present data revealed that the tested product of 21.6 g protein and 21.1 g carbohydrates can be classified as LGI and LGL. Given the multiple health effects, the tested food could potentially be part of a health promoting diet for both patients and healthy individuals.
